# What kind of sick person would see a Foot Doctor! The Foot Disease in Inpatients Study (FDIS)

**DOI:** 10.1186/1757-1146-8-S2-O24

**Published:** 2015-09-22

**Authors:** Peter A Lazzarini, Vanessa Ng, Suzanne S Kuys, Maarten C Kamp, Michael C d'Emden, Courtney Thomas, Jude Wills, Ewan M Kinnear, Scott Jen, Sheree E Hurn, Lloyd Reed

**Affiliations:** 1 School of Clinical Sciences, Queensland University of Technology, Brisbane, Queensland, 4059, Australia; 2 Allied Health Research Collaborative, Metro North Hospital & Health Service, Queensland Health, Brisbane, Queensland, 4032, Australia; 3 Department of Podiatry, Metro North Hospital & Health Service, Queensland Health, Brisbane, Queensland, 4032, Australia; 4 Musculoskeletal Research Program, Griffith Health Institute, Griffith University, Gold Coast, Queensland, 4222, Australia; 5 Department of Endocrinology and Diabetes, Royal Brisbane and Womens Hospital, Brisbane, Queensland, 4029, Australia; 6 School of Medicine, The University of Queensland, Brisbane, Queensland, 4072, Australia; 7 Department of Podiatry, North West Hospital & Health Service, Mount Isa, Queensland, 4825, Australia; 8 Department of Podiatry, Central Queensland Hospital & Health Service, Rockhampton, Queensland, 4700, Australia; 9 Department of Podiatry, West Moreton Hospital & Health Service, Queensland Health, Ipswich, Queensland, 4305, Australia

**Keywords:** Foot, inpatients, disease, complications, health profesional

## Background

Many different guidelines recommend people with foot complications, or those at risk, should attend multiple health professionals for foot care each year. However, few studies have investigated the characteristics of those attending health professionals for foot care and if those characteristics match those requiring foot care as per guideline recommendations. The aim of this paper was to determine the associated characteristics of people who attended a health professional for foot care in the year prior to their hospitalisation.

## Methods

Eligible participants were all adults admitted overnight, for any reason, into five diverse hospitals on one day; excluding maternity, mental health and cognitively impaired patients. Participants underwent a foot examination to clinically diagnose different foot complications; including wounds, infections, deformity, peripheral arterial disease and peripheral neuropathy. They were also surveyed on social determinant, medical history, self-care, foot complication history, and, past health professional attendance for foot care in the year prior to hospitalisation.

## Results

Overall, 733 participants consented; mean(±SD) age 62(±19) years, 408 (55.8%) male, 172 (23.5%) diabetes. Two hundred and fifty-six (34.9% (95% CI) (31.6-38.4)) participants had attended a health professional for foot care; including attending podiatrists 180 (24.5%), GPs 93 (24.6%), and surgeons 36 (4.9%). In backwards stepwise multivariate analyses attending any health professional for foot care was independently associated (OR (95% CI)) with diabetes (3.0 (2.1-4.5)), arthritis (1.8 (1.3-2.6)), mobility impairment (2.0 (1.4-2.9)) and previous foot ulcer (5.4 (2.9-10.0)). Attending a podiatrist was independently associated with female gender (2.6 (1.7-3.9)), increasing years of age (1.06 (1.04-1.08), diabetes (5.0 (3.2-7.9)), arthritis (2.0 (1.3-3.0)), hypertension (1.7 (1.1-2.6) and previous foot ulcer (4.5 (2.4-8.1). While attending a GP was independently associated with having a foot ulcer (10.4 (5.6-19.2).

## Conclusions

Promisingly these findings indicate that people with a diagnosis of diabetes and arthritis are more likely to attend health professionals for foot care. However, it also appears those with active foot complications, or significant risk factors, may not be more likely to receive the multi-disciplinary foot care recommended by guidelines. More concerted efforts are required to ensure all people with foot complications are receiving recommended foot care.

